# Cannabinoid receptor 2-63 RR variant is independently associated with severe necroinflammation in HIV/HCV coinfected patients

**DOI:** 10.1371/journal.pone.0181890

**Published:** 2017-07-31

**Authors:** Caterina Sagnelli, Caterina Uberti-Foppa, Hamid Hasson, Giulia Bellini, Carmine Minichini, Stefania Salpietro, Emanuela Messina, Diletta Barbanotti, Marco Merli, Francesca Punzo, Nicola Coppola, Adriano Lazzarin, Evangelista Sagnelli, Francesca Rossi

**Affiliations:** 1 Department of Mental Health and Public Medicine, Section of Infectious Diseases, University of Campania Luigi Vanvitelli, Naples, Italy; 2 Department of Infectious Diseases, Vita-Salute University, San Raffaele Scientific Institute, Milan, Italy; 3 Department of Pediatrics, University of Campania Luigi Vanvitelli, Naples, Italy; Centers for Disease Control and Prevention, UNITED STATES

## Abstract

**Objective:**

This is the first study to analyze the impact of the rs35761398 variant of the CNR2 gene leading to the substitution of GLN (Q) of codon 63 of the cannabinoid receptor 2 (CB2) with ARG (R) on the clinical presentation of chronic hepatitis in HIV/HCV coinfected patients.

**Methods:**

Enrolled in this study were 166 consecutive HIV/HCV coinfected patients, naïve for HCV treatment. A pathologist unaware of the patients’ condition graded liver fibrosis, necroinflammation (Ishak) and steatosis. All patients were screened for the CB2 rs35761398 polymorphism.

**Results:**

Of the 166 HIV/HCV coinfected patients, 72.9% were males, 42.5% were infected with HCV-genotype-3 and 60.2% had been intravenous drug users. The median age was 40.6 years and the immunological condition good (median CD4^+^ cells/mm^3^ = 507, IQR: 398.0–669.5). Thirty-five (21.1%) patients were naive for ART and 131(78.9%) were on ART. The CB2-RR variant was detected in 45.8% of patients, QR in 38.6% and QQ in 15.7%. Patients with CB2-RR showed a necroinflammation score (HAI) ≥9 more frequently than those with CB2-QQ or CB2-QR (32.9% vs. 11.5% and 14.1%, respectively, *p*≤0.001). In the multivariate analysis, the CB2-RR variant (*p* = 0.03) and liver fibrosis were both identified as independent predictors of the entity of liver necroinflammation (*p* = 0.0001).

**Conclusion:**

This study shows interesting interplay between the CB2-RR variant and liver necroinflammation in chronic hepatitis patients with HIV/HCV coinfection, an observation of clinical value that coincides with the interest in the use of the CB2 agonists and antagonists in clinical practice emerging from the literature.

## Introduction

One-third of HCV-monoinfected patients with chronic hepatitis C (CHC) progress to cirrhosis in approximately 20 years [1], the risk factors for higher rates and more rapid transition being an older age, alcohol abuse, male sex, and human immunodeficiency virus (HIV) coinfection [2–21].

Several other factors have been found to be associated with CHC progression and severity, such as the duration of HCV infection, coinfection with hepatitis B virus (HBV), insulin resistance, diabetes, high body mass index (BMI), immunosuppression of different etiology, alcohol abuse, drug addiction, interleukin-28B polymorphism and the cannabinoid receptor 2 (CB2)-63 variants [22–28]. The CB2-63 RR variant was identified as an independent predictor of some aggressive autoimmune pathologies [29,30] and of some immune mediated diseases associated with HCV chronic carriers [31].

Two types of CB are known, type 1 (CB1) and type 2 (CB2), which exert mainly anti-inflammatory and immunomodulatory action [32]. CB2 is expressed predominantly in the cells of the immune system [33,34], and particularly in CD4+ cells [35], Kupffer cells and hepatic stellate cells, which all play an essential role in the acute and chronic responses to toxic and infectious agents [36]. The CB2 expression in hepatocytes is induced by various inflammatory processes, such as that induced by HCV replication on the intracellular lipid membrane [33,34].

CB2 may function as a chemotactic modulator that inhibits CXCR4-induced chemotaxis in trafficking T cells [37] and the chemokine receptor 5 (CCR5), a co-receptor for HIV-1 cell entry. By interfering with the action of other chemoattractants [38], as well as the chemotaxis of immune cells, and endothelium and leukocyte infiltration, cannabinoids may reduce the inflammatory processes and injury to the endothelial barriers, such as the blood brain barrier, and HIV-1 infection of susceptible cells [39].

It has also been suggested that in HIV infection, endocannabinoids may interact with different pro-inflammatory events influencing HIV-1 pathogenesis [39], downregulating CCR5 and inhibiting viral expression [40].

A polymorphism at codon 63 of the CB2 gene allows the substitution of glutamine, GLN (Q), with arginine, ARG (R), with a consequent differentiation in the protein polarization. These CB2 variants affect differently the ability of CB2 to perform its inhibitory function [33,41].

To our best knowledge, the present paper is the first to analyze the impact of the rs35761398 variant of the CNR2 gene on the clinical history of biopsy proven chronic hepatitis in HIV/HCV coinfected patients.

We investigated the impact of the rs35761398 variant of the CNR2 gene on the clinical presentation of biopsy proven chronic hepatitis in 166 HIV/HCV coinfected patients.

## Material and methods

### Ethics statement

All procedures applied in the study were in accordance with the international guidelines, with the standards of human experimentation of the local Ethics Committees and with the Helsinki Declaration of 1975, revised in 1983. At the time of the first observation, each patient signed their informed consent to undergo liver biopsy, for the collection and storage of serum and whole blood and for the collection and use in clinical research of the data obtained, as established by the Ethics Committee of the Azienza Ospedaliera Universitaria—University of Campania Luigi Vanvitelli, Naples, previously named Second University of Naples (protocol number 112/15 March 2013).

### Patients

One-hundred and sixty-six consecutive anti-HIV-positive patients with HCV-RNA-positive CHC were enrolled from 1993 to 2008 at the time they underwent their first liver biopsy at one of the two participating Units of Infectious Diseases, one in Milan and the other in Naples, after an observation period of at least 18 months. These two centers have cooperated for several years in numerous clinical investigations applying the same clinical and laboratory approach [14,16,42–44].

All patients enrolled were HBsAg-negative, declared no active alcohol abuse (≥ 40 g/day for males and ≥ 30 g/day for females for at least 5 years) or statin intake and had no evidence of autoimmune or genetic disorders that may induce liver disease. These patients were naive for anti-HCV treatment and none showed ultrasound or serological signs of HCC. None of the 166 patients enrolled had impaired fasting, glycemia or diabetes and had never received anti-tuberculosis or antifungal medications.

At the time of enrolment, 131 (78.2%) patients were receiving antiretroviral therapy (ART) and 35 had been left untreated in accordance with the current DHHS guidelines [45].

Samples of serum and whole blood were obtained at the time the liver biopsy was performed, stored at −80°C and never thawed until used for this investigation.

### Liver biopsy

Percutaneous liver biopsy (LB) [46] was performed for all patients under US guidance using a disposable modified Menghini needle (16 gauge—external diameter 1.65 mm). A liver specimen of more than 1.5 cm in length was always obtained and at least 11 portal tracts were examined in each sample. Specimens were fixed in formalin, embedded in paraffin and stained with hematoxylin-eosin and Masson’s trichrome stain. Liver biopsies were examined by a skilled pathologist who, blinded for the clinical and laboratory data, used the Ishak scoring system to grade necroinflammation and fibrosis [47]. In the absence of a standardized scoring system to assess liver steatosis, we used a homemade scoring system obtained with a partial modification of Kleiner’s scoring system for NAFLD [48], assigning score 1 to a fatty deposition in 1–10% of hepatocytes, score 2 in 11–31%, score 3 in 31–60% and score 4 in >60%.

### Serological determinations

Serum HBsAg was sought by a commercial immunoenzymatic assay (Abbott Laboratories, North Chicago, IL, USA) and the anti-HCV antibody by a 3^rd^ generation commercial immunoenzymatic assay (Ortho Diagnostic Systems, Neckargemund, Germany). Antibodies to HIV 1 and 2 were sought using a commercial ELISA (Abbott Lab., North Chicago, Ill, USA), and positive results were always confirmed by Western blot (Genelabs Diagnostics, Science Park Drive, Singapore), in accordance with the Italian guidelines.

HCV RNA was quantified by a real-time polymerase chain reaction (PCR) in a Light cycler 1.5 (Roche Diagnostics, Branchburg, NJ, USA); by this method, the detection limit in plasma samples is estimated at around 40 IU/mL. HCV genotyping was performed by a Line-Probe assay (INNO-LIPA HCV-II; Innogenetics, Zwigndrecht, Belgium). The HIV viral load was assessed using the Amplicor HIV Monitor 1 test (Roche Molecular Systems Inc., Branchburg, New Jersey).

Lymphocyte subsets (CD4+, CD8+) were evaluated by flow cytofluorimetry using monoclonal antibodies and a fluorescence-activated cell sorter scan (Becton Dickinson, Mountain View, USA). Liver function tests and triglyceride and cholesterol assessment were carried out applying routine methods. The body mass index (BMI: kg/m^2^) was determined by standard procedure.

### The CNR2 rs35761398 polymorphism

Genom ic DNA was extracted from peripheral whole blood with a DNA extraction kit (Roche Diagnostics, Branchburg, NJ, USA) after written informed consent. Molecular screening for the CNR2 rs35761398 polymorphism (CAA/CGG) underlying the CB2Q63R substitution was performed using a TaqMan Assay (Real Master Mix Probe, 5 PRIME, Germany). Primers and probes were the following: sense primer 59-GTGCTCTATCTGATCCTGTC- 39 and anti-sense primer 59-TAGTCACGCTGCCAATC-39; AA-probe 59-CCCACCAACTCCGC- 39 and GG-probe 59-CCCACCGGCTCCG-39 (PRIMM, Milan, Italy). Both PCR and post-PCR allelic discrimination were carried out on an ABI PRISM 9600 apparatus (Applied Biosystems, Foster City, CA). Random samples were confirmed by direct PCR sequencing consisting of 94°C for 4 min followed by 31 cycles of 94°C for 30 s, 60°C for 30 s and 72°C for 30 s with forward 59-GAGTGGTCCCCAGAAGACAG-39 and reverse 59-CACAGAGGCTGTGAAGGTCA-39 primers. PCR products were analyzed using an ABI PRISM 3100 automated sequencer (Applied Biosystems, Foster City, CA) and the Big Dye Terminator reaction kit (Applera, Foster City, CA), according to the manufacturer’s instructions. All primers were chosen using Primer3 software (http://primer3.sourceforge.net/). This procedure had been used in previous investigations [49,50].

### Statistical analysis

The patients’ allele frequencies were tested for the Hardy-Weinberg equilibrium with Fisher’s exact test.

Continuous variables not normally distributed were summarized as median and interquartile range (IQR), and categorical variables as absolute and relative frequencies. Mood’s Median test was used to compare continuous variables, and the chi-square test for categorical variables. A *p* value < 0.05 was considered significant.

Multivariate logistic regression analysis was performed to explore the overall effect of the parameters significantly associated with the risk of a histological activity index (HAI) ≥9 at the univariate analysis.

The statistical analysis was performed using Statgraphics Centurion XV.II (Adalta, Arezzo, Italy; Statpoint Technologies Inc., Virginia, USA).

## Results

Of the 166 HIV/HCV coinfected patients enrolled in the present study, 72.9% were males, 42.5% were infected with HCV-genotype-3 and 38.1% with genotype 1, 60.2% had a history of previous intravenous drug use (IVDU) but none stated active drug addiction. The median age was 40.5 years and the immunological condition good on the basis of the median CD4^+^ cells/mm^3^ count: 507.0 (IQR: 398.0–669.5) at enrolment with a nadir of 258.0 (IQR: 163.5–404.5). Thirty-five (21.1%) patients were naive for ART and 131 (78.9%) were on ART. Untreated patients showed higher CD4^+^ cell counts than those on ART: 581.0 cells/ mm^3^ vs. 492.0 cells/ mm^3^ (*p* = 0.01) at enrolment and 431.0 cells/ mm^3^ vs. 230.5 cells/ mm^3^ (*p* = 0.01) nadir values.

The CB2-63 RR variant was detected in 45.8% of the patients, QR in 38.5% and QQ in 15.7%. The minor allele frequency was QR and the genotypes were distributed according to the Hardy–Weinberg equilibrium. The mean grade of liver necroinflammation, expressed as HAI scores ranging from 0 to 18, was 5.4 ± 3.0 (SD), the mean stage of liver fibrosis was 2.3 ± 1.6 (range 0–4) and the mean degree of liver steatosis 1.7 ± 1.3 (range 0–4).

### Demographic, biochemical and histological characteristics of the patients according to the CB2-63 genotype variants

The 64 subjects with the CB2-63 QR variant and the 76 with the CB2-63 RR variant more frequently than the 26 patients with the CB2-63 QQ variant had a history of previous IVDU (67.2% and 58.0% vs. 50.0%, respectively) (Table 1). Patients with CB2-RR showed a moderate or severe HAI score (9–18) more frequently than those with CB2-63 QQ or CB2-63 QR (32.9% vs. 11.5% and 14.1%, respectively, *p* = 0.01; Odds Ratio 3.18627; 95% CL 1.47008–6.90597). No other significant difference was observed in the demographic, laboratory or histological data, nor in ART treatment. (Table 1)

**Table 1:** Demographic, biochemical and histological characteristics of the 166 HIV/HCV coinfected patients according to the CB2 63 genotype variants.

	CB2 QQ	CB2 QR	CB2 RR	p-value
**N° of patients**	26	64	76	
**Males, N° (%)**	23 (88.5)	41 (64.1)	57 (75.0)	0.05
**Age, years, median (IQR)**	40.6 (36.4–43.4)	40.0 (37.1–42.6)	42.0 (37.98–46.6)	0.06
**BMI, m2/kg, median (IQR)**	23.3 (21.7–25.4)	22.5 (21.1–24.2)	23.2 (21.5–25.3)	0.07
**Past IVDU, N°(%)**	13 (50.0)	43 (67.2)	44 (58.0)	0.27
**Nadir of CD4+, cell/mmc, median (IQR)**	231.0 (82.5–322.5)	261.5 (160.3–427.0)	283.0 (182.5–378.8)	0.20
**HIV RNA cps/mL, median (IQR)**	10870.0 (2450.5–44338.0)	3800.0 (1007.0–18687.0)	13301.0 (3991.0–37219.0)	0.37
**CD4, cell/mmc, median (IQR)**	474.0 (369.5–557.3)	570.0 (420.3–725.3)	492.0 (394.8–659.0)	0.56
**AST, IU/mL, median (IQR)**	58.0 (41.75–71.00)	56.0 (39.5–87.8)	67.0 (40.0–132.0)	0.04
**ALT, IU/mL, median (IQR)**	89.5 (58.3–127.0)	73.5 (44.0–114.0)	111.5 (49.0–158.8)	0.17
**Bilirubin, mg/dL, median (IQR)**	0.7 (0.53–0.92)	0.72 (0.5–0.5)	0.7 (0.47–1.0)	0.91
**GGT, IU/mL, median (IQR)**	87.0 (42.75–157.50)	70.5 (38.0–154.25)	75.0 (36.0–189.0)	0.13
**ALP, IU/mL, median (IQR)**	174.0 (133.0–240.5)	198.0 (139.0–253.0)	187.5 (136.50–249.8)	0.39
**Glucose, mg/dL, median (IQR)**	95.0 (80.5–104.50)	85.50 (79.0–92.8)	88.0 (81.0–97.5)	0.13
**Creatinine, mg/dL, median (IQR)**	0.79 (0.1–0.84)	0.8 (0.7–0.9)	0.8 (0.7–0.9)	0.83
**Triglycerides, mg/dL, median (IQR)**	125.5 (98.75–153.0)	122.0 (81.3–153.0)	133.0 (88.5–184.0)	0.77
**Total cholesterol, mg/dL, median (IQR)**	170.5 (149.75–195.0)	160.0 (129.0–186.0)	162.0 (129.5–194.8)	0.75
**HCV RNA, IUx103, median (IQR)**	445956.0 (115463.3–1575000.0)	452925.0 (104103.0–1771436.5)	730500.0 (336750.0–1411766.5)	0.76
**Duration of HIV infection, years, median (IQR)**	15.98 (10.95–17.4)	14.4 (7.7–18.1)	11.7 (6.86–18.5)	0.19
**ART-treated, N°(%)**	22 (8.05)	52 (81.3)	57 (75.0)	0.49
**ART-naïve, N°(%)**	4 (15.0)	12 (18.7)	19 (25.0)	
**Duration of ART, years, median (IQR)**	6.5 (3.4–12.8)	7.7 (5.9–10.4)	8.22 (6.28–11.2)	0.41
**HCV genotype 1, N° (%)**	10 (40.0)	22 (35.5)	29 (39.7)	0.89
**HCV genotype 2, N° (%)**	1 (4.0)	2 (3.3)	5 (6.9)	
**HCV genotype 3, N° (%)**	10 (40.0)	27 (43.55)	31 (42.5)	
**HCV genotype 4, N° (%)**	4 (16.0)	11 (17.7)	8 (10.96)	
**HCV genotype missing, N°**	1	2	3	
**Liver histology:**				
**HAI, score (M ± SD)**	5.8 ± 2.6	5.3 ± 2.7	6.8 ± 3.4	0.056
**HAI score 0–8, N° (%)**	23 (88.5)	55 (85.9)	51 (67.1)	0.01
**HAI score 9–18, N° (%)**	3 (11.5)	9 (14.1)	25 (32.9)	
**Fibrosis, score (M ± SD)**	2.4 ± 1.4	2.2 ± 1.7	2.4 ± 1.6	0.75
**Fibrosis score 0–3, N° (%)**	22 (84.6)	53 (82.8)	61 (80.3)	0.86
**Fibrosis score 4–6, N° (%)**	4 (15.4)	11 (17.2)	15 (19.7)	
**Steatosis, score (M ± SD)**	1.9 ± 1.4	1.7 ± 1.3	1.6 ± 1.3	0.57
**Steatosis score 0–2, N° (%)**	15 (57.7)	45 (70.0)	50 (65.8)	0.51
**Steatosis score 3–4, N° (%)**	11 (42.3)	19 (30.0)	26 (34.2)	

### Patients’ characteristics according to the grade of liver necroinflammation

Table 2 shows a comparative analysis of the initial characteristics of the 166 patients according to the degree of liver necroinflammation, absent or mild (HAI score 0–8) versus moderate or severe (HAI score 9–18). Compared with the 129 patients with lower HAI scores, the 37 patients with moderate or severe necroinflammation more frequently had the CB2-RR variant (65.6% vs. 47.3%, *p* = 0.01) and showed higher AST (112.0 vs. 54.0, *p* = 0.000001), ALT (137.0 vs. 72.0, *p =* 0.0004), and ALP (205.0 vs. 179.0, *p* = 0.008) serum levels and higher liver fibrosis scores (3.6 ± 1.5 vs 1.9 ± 1.4, *p*< 0.0001, and steatosis scores (2.0 ± 1.3 vs 1.6 ± 1.3, *p* = 0.03) (Table 2). In particular, patients with a moderate or high HAI score, compared to those with a lower score, more frequently showed severe fibrosis score (stage 4–6 in 40.5% vs 11.6%, *p* = 0.0001) and severe steatosis score (grade 3–4 in 48.7% vs. 29.5%) (Table 2). No other differences in the two necroinflammation subgroups reached statistical significance (Table 2), nor in ART treatment.

**Table 2:** Demographic, biochemical and histological characteristics of the 166 HIV/HCV coinfected patients according to necroinflammation (HAI score).

	HAI score 0–8	HAI score 9–18	p-value
**N° of patients**	129	37	
**Males, N° (%)**	91 (70.5)	30 (81.1)	0.20
**Age, years, median (IQR)**	40.9 (37.2–44.2)	41.0 (38.0–44.3)	0.85
**BMI, m2/kg, median (IQR)**	22.8(21.2–24.86)	23.6 (21.5–25.4)	0.72
**Nadir of CD4**^+^**, cell/mmc, median (IQR)**	258.0 (165.25–422.0)	272.0 (149.0–374.0)	0.82
**Past IVDU, N°(%)**	77 (59.6)	23 (72.2)	0.93
**HIV RNA, cps/mL, median (IQR)**	8321.0 (1541.5–36329.8)	12000.0 (3183.5–31052.0)	0.19
**HIV RNA Negative (< 50 cps /mL), N° (%)**	55 (42.6)	11 (35.1)	0.16
**HIV RNA Positive (≥ 50 cps /mL), N° (%)**	65 (50.4)	23 (62.2)	
**CD4**^+^**, cell/mmc, median (IQR)**	509.5 (404.5–691.0)	466.0 (355.0–608.5)	0.37
**CD4**^+^ ≤ **500 cell/mmc, N° (%)**	59 (45.7)	21 (56.8)	0.25
**CD4**^+^ **> 500 cell/mmc, N° (%)**	69 (53.5)	16 (43.2)	
**AST, IU/mL, median (IQR)**	54.0 (38.3–77.0)	112.0 (76.0–166.5)	0.0000016
**ALT, IU/mL, median (IQR)**	72.0 (43.5–120.0)	137.0(87.5–223.5)	0.00039
**Bilirubin, mg/dL, median (IQR)**	0.7 (0.49–1.05)	0.8 (0.4–1.1)	0.7
**GGT, IU/mL, median (IQR)**	68.5 (33.0–155.3)	99.0 (60.0–189.0)	0.06
**ALP, IU/mL, median (IQR)**	179.0 (132.0–238.3)	205.0 (177.0–274.0)	0.008
**Glucose, mg/dL, median (IQR)**	87.0 (79.0–95.0)	91.0 (83.3–99.8)	0.0153
**Creatinine, mg/dL, median (IQR)**	0.78 (0.7–0.9)	0.8 (0.7–0.8)	0.64
**Triglycerides, mg/dL, median (IQR)**	127.0 (86.5–169.8)	123.0 (169.75–182.0)	1
**Total cholesterol, mg/dL, median (IQR)**	163.0 (137.0–194.0)	154.0 (121.5–180.5)	0.53
**HCV RNA, IUx10**^**3**^**, median (IQR)**	511250.0 (115550.0–1432735.0)	732000 (342000.0–1487013.0)	0.30
**Duration of HIV infection, years, median (IQR)**	14.4 (7.7–18.1)	13.3 (7.5–18.3)	0.37
**ART-treated, N°(%)**	103 (82.2)	28 (75.7)	0.58
**ART-naïve, N°(%)**	26 (20.2)	9 (24.3)	
**Duration of ART, years, median (IQR)**	8.2 (6.2–11.3)	6.79 (4.46–10.3)	0.2
**CB2-63 QQ, N° (%)**	23 (17.8)	3 (8.1)	0.01
**CB2-63 QR, N° (%)**	55 (42.6)	9 (24.3)	
**CB2-63 RR, N° (%)**	51 (47.3)	25 (65.6)	
**HCV genotype 1, N° (%)**	47 (38.2)	14 (37.8)	0.19
**HCV genotype 2, N° (%)**	7 (5.7)	1 (2.7)	
**HCV genotype 3, N° (%)**	48 (39.0)	20 (54.0)	
**HCV genotype 4, N° (%)**	21 (17.1)	2 (5.4)	
**HCV genotype missing, N°**	6	0	
**Liver histology:**			
**Fibrosis, score (M ± SD)**	1.9± 1.4	3.6± 1.5	0.00000068
**Fibrosis score 0–3, N° (%)**	114 (88.4)	22 (67.6)	0.0001
**Fibrosis score 4–6, N° (%)**	15 (11.6)	15 (40.5)	
**Steatosis, score (M ± SD)**	1.6 ± 1.3	2.0 ± 1.3	0.03
**Steatosis score 0–2, N° (%)**	91 (70.5)	19 (51.3)	0.03
**Steatosis score 3–4, N° (%)**	38 (29.5)	18 (48.7)	

### Multivariate analysis

The association between the CB2-63 RR variant and HAI score 9–18 was analyzed in a multivariate analysis (Table 3) including as covariates the age at enrolment, HIV RNA (≤ 100 vs. > 100 copies/ml), CD4+cells/mL count (≤ 500 vs. > 500 cells/mL), liver fibrosis stage (absent or mild vs. moderate or severe; 0–3 vs. 4–6) and ART regimen (yes vs. no). Homozygous Q63 and heterozygous QR patients were assessed together since the CB2 Q63R variant exerts a significant effect on modulation of necroinflammation only in homozygosity for the R63-encoding allele. The CB2-63 RR variant (*p* = 0.02) and severe fibrosis (*p* = 0.001) were both found to be independently associated with severe necroinflammation, with a correlated risk of developing severe necroinflammation about three-fold greater in R63 homozygous carriers compared to QQ and QR carriers.

**Table 3:** Logistic regression for HAI score ≥9 for the 166 HIV/HCV coinfected patients.

Parameter	Estimate	Standard Error	Estimated Odds Ratio	Chi-Square	Df	P-Value
**CONSTANT**	-2.536	3.162				
**Age, years**	-0.073	0.044	0.930	2.743	1	0.09
**Gender (F vs. M)**	-0.365	0.658	0.694	0.312	1	0.57
**BMI, m2/kg**	-0.001	0.088	0.999	0.000	1	0.99
**CB2-63 (RR vs. QR + QQ)**	1.077	0.492	**2.936**	4.978	1	**0.026**
**Fibrosis stage (4–6 vs. 0–3)**	1.580	0.533	**4.856**	10.876	1	**0.003**
**Steatosis score (3–4 vs. 0–2)**	0.149	0.515	1.161	0.083	1	0.77
**AST, IU/mL**	0.017	0.007	**1.017**	7.334	1	**0.007**
**ALT, IU/mL**	-0.002	0.088	0.999	0.252	1	0.61
**ALP, IU/mL**	0.002	0.003	1.002	0.646	1	0.42
**Glucose, mg/dL**	0.017	0.013	1.017	1.732	1	0.19

## Discussion

The present study is the first, to the best of our knowledge, to demonstrate that the CB2-63 RR variant is an independent predictor of moderate/severe liver necroinflammation in chronic hepatitis patients with HIV/HCV coinfection. In accordance, the CB2-63 RR variant was recently found to be independently associated with some aggressive autoimmune pathologies such as celiac disease and childhood immune thrombocytopenic purpura [29,30] and with immune mediated diseases in HCV chronic carriers [31]. In addition, the endocannabinoid system has been demonstrated to play a role in several systemic inflammatory disorders such as rheumatoid arthritis [51]. It has also been reported that in obese children with ultrasound-proven liver steatosis, the ALT serum level is significantly higher in children with the QR or RR variant than in those with the CB2-63 QQ variant [52].

In our previous study performed on 253 anti-HIV negative subjects with HCV chronic infection the CB2-63 QQ variant and an older age were found to be independently associated with an asymptomatic, inactive condition characterized by persistently normal alanine-aminotransferase (PNALT) values at a two-monthly determination for 18 months or more [50], whereas in a subsequent investigation on 169 anti-HIV-negative patients with HCV-related chronic hepatitis, the CB2-63 QQ variant was associated with more severe liver necroinflammation [53]. Based on the observation that CB2-mediated inhibition of T-cell proliferation has been shown to be normal in T cells deriving from QQ subjects and reduced two-fold in T cells from subjects with the RR homozygous variant [54], these associations were interpreted as the result of differences in the strength and duration of the inhibition of the T cells expressing CB2-63 QQ or CB2-63 RR and of the consequent lesser or more vigorous immune response against infected hepatocytes. Interestingly, the mean age of the 53 PNALT subjects in the first study was higher than that of the second study (58.5 vs 49.6 years), suggesting that a substantial percentage of PNALT subjects may have reached this status after a variety of immunological conditions ranging from a strong cellular immune response in the HCV acute infection and the initial phase of chronic hepatitis to a failure of the cellular immune response in the more advanced inactive phase of the illness [53].

The anti-HIV-positive patients in the present study were much younger (mean age 40.5 years) than those with chronic HCV monoinfection (PNALT subjects or chronic hepatitis patients) mentioned above and plausibly exposed to HCV and its associated immunological pressure for a shorter time, a difference that might influence the expression of the CB2-63 variants. Noteworthy, HIV replication is responsible for a variety of immunological reactions favouring more severe HCV-related necroinflammation [53] and may interact with the CB2-63 genetic expression. Indeed, the specific activation of CB2 receptors may inhibit not only the production of autoantibodies, proinflammatory cytokines and matrix metalloproteinases, but also bone erosion, an immune response mediated by T cells and the proliferation of fibroblast-like synoviocytes [51]. In addition, CB2 exerts an inhibitory effect on inflammatory processes [55], including macrophage migration [56], and provides an important therapeutic target for reducing/ablating some immunopathological processes associated with HIV-1 infection [56], a beneficial effect confirmed by the observation that CBR2 agonists reduce AIDS symptoms [57].

The activation of CB2 inhibits CXCR4-tropic HIV infection by altering the CD4+ T-cell actin dynamic and reduces the frequency of infected cells by 30–60% [57]. The level of CB2 activation able to inhibit the HIV virus does not actually alter the CXCR4 surface expression, but significantly reduces CXCR4-mediated G-protein binding and downstream signaling [57], factors accompanied by a reduction in F-actin accumulation [58] and the prevention of the cortical actin rearrangements required for reverse transcription and migration of the viral preintegration complex into the nucleus [58]. Taken together, these data suggest that CB2 cross-regulates CXCR4 and that this inhibitory cross-talk may decrease HIV infection [57]. Therefore, the reciprocal effects exerted by HIV infection and the CB2 receptor may explain the different association of severe liver necroinflammation and the CB2 variants: CB2 RR in HIV/HCV coinfected patients who show an aggressive disease with a rapid progression to liver cirrhosis and hepatocellular carcinoma and CB2 QQ in HCV-monoinfected patients with an asymptomatic indolent course of the liver disease.

Concluding on this point, the data from this study identified the CB2 RR variant as an independent predictor of more severe liver necroinflammation in HIV/HCV coinfected patients with chronic hepatitis.

The number of patients investigated in the present study may be considered barely sufficient for a genetic association, a limitation offset by the gold-standard method used to detect liver lesions (liver biopsy examination by a skilled pathologist) and by the novelty of the data reported. In addition, *in-vitro* studies are needed to ascertain how the CB2-63 variants modify the mechanism of liver cell injury in HCV infection, both in HIV-infected and non-infected patients. Further clinical investigations on this topic are welcome, particularly because of the increasing interest in the use of CB2 agonists and antagonists in clinical practice.

## Abbreviations

ALP: alkaline phosphatase
ALT: alanine aminotransferase
ART: antiretroviral therapy
AST: aspartate aminotransferase
BMI: body mass index
CB2: cannabinoid receptor 2
CHC: chronic hepatitis C
CI: confidence interval
GGT: gamma glutamyl transferase
HAART: highly active antiretroviral therapy
HAI: histological activity index
HCC: hepatocellular carcinoma
HCV: hepatitis C virus
HIV: human immunodeficiency virus
IQR: interquartile range
M: Mean
OR: odds ratio
SD: standard deviation
